# Neutralizing Monoclonal Anti-SARS-CoV-2 Antibodies Isolated from Immunized Rabbits Define Novel Vulnerable Spike-Protein Epitope

**DOI:** 10.3390/v13040566

**Published:** 2021-03-26

**Authors:** Efi Makdasi, Yinon Levy, Ron Alcalay, Tal Noy-Porat, Eran Zahavy, Adva Mechaly, Eyal Epstein, Eldar Peretz, Hila Cohen, Liat Bar-On, Theodor Chitlaru, Ofer Cohen, Itai Glinert, Hagit Achdout, Tomer Israely, Ronit Rosenfeld, Ohad Mazor

**Affiliations:** Israel Institute for Biological Research, Ness-Ziona 74100, Israel; efim@iibr.gov.il (E.M.); yinonl@iibr.gov.il (Y.L.); rona@iibr.gov.il (R.A.); taln@iibr.gov.il (T.N.-P.); eranz@iibr.gov.il (E.Z.); advam@iibr.gov.il (A.M.); eyale@iibr.gov.il (E.E.); eldarp@iibr.gov.il (E.P.); hilac@iibr.gov.il (H.C.); liatb@iibr.gov.il (L.B.-O.); theodorc@iibr.gov.il (T.C.); oferc@iibr.gov.il (O.C.); itaig@iibr.gov.il (I.G.); hagita@iibr.gov.il (H.A.); tomeri@iibr.gov.il (T.I.)

**Keywords:** COVID-19, SARS-CoV-2, neutralizing antibody, monoclonal antibody, single-cell sort, spike, rabbit immunization

## Abstract

Monoclonal antibodies represent an important avenue for COVID-19 therapy and are routinely used for rapid and accessible diagnosis of SARS-CoV-2 infection. The recent emergence of SARS-CoV-2 genetic variants emphasized the need to enlarge the repertoire of antibodies that target diverse epitopes, the combination of which may improve immune-diagnostics, augment the efficiency of the immunotherapy and prevent selection of escape-mutants. Antigen-specific controlled immunization of experimental animals may elicit antibody repertoires that significantly differ from those generated in the context of the immune response mounted in the course of disease. Accordingly, rabbits were immunized by several recombinant antigens representing distinct domains of the viral spike protein and monoclonal antibodies were isolated from single cells obtained by cell sorting. Characterization of a panel of successfully isolated anti-receptor binding domain (RBD) and anti-N-terminal domain (NTD) antibodies demonstrated that they exhibit high specificity and affinity profiles. Anti-RBD antibodies revealing significant neutralizing potency against SARS-CoV-2 in vitro were found to target at least three distinct epitopes. Epitope mapping established that two of these antibodies recognized a novel epitope located on the surface of the RBD. We suggest that the antibodies isolated in this study are useful for designing SARS-CoV-2 diagnosis and therapy approaches.

## 1. Introduction

Since its onset, in the beginning of 2020, the coronavirus disease 19 (COVID-19) pandemic, caused by the severe acute respiratory syndrome coronavirus 2 (SARS-CoV-2), unprecedentedly affected global public health, economy and society. Accordingly, the scientific community is dedicating massive efforts towards developing effective vaccines, therapeutic countermeasures and diagnostic methods.

SARS-CoV-2 utilizes the envelope homo-trimeric spike glycoprotein (S) as a major route for cellular infection [1,2]. The SARS-CoV-2 S protein (1273 amino acid residues) is composed of two distinct subunits, S1 and S2. The S1 subunit includes the receptor binding domain (RBD), known to specifically bind the human angiotensin-converting enzyme 2 (ACE2) receptor on host cell surfaces. The S2 subunit mediates the fusion of the viral and cellular membranes, leading to viral entry [2,3]

We and others have recently documented the successful use of therapeutic monoclonal antibodies (mAbs) for COVID-19 in several animal models [4,5,6,7,8,9,10,11] and humans [12,13,14]. Most of the highly neutralizing antibodies reported against SARS-CoV-2 were derived from convalescent individuals and shown to target the RBD [15,16,17,18,19]. Recently, vulnerable epitopes located within non-RBD regions were also shown to be the target of several SARS-CoV-2 neutralizing antibodies. Specifically, the N-terminal domain (NTD) of the S1 subunit [8,20,21,22] and the S2 region [23] were suggested as an alternative and/or complementary target for neutralizing antibodies. It was recently reported that SARS-CoV-2 undergoes and accumulates genetic mutations, some of which hamper the ability of neutralizing antibodies to bind the virus [24]. Thus, there is a need to expand the repertoire of antibodies that target different epitopes, either for therapeutic purposes or towards the development of specific immunodiagnostic assays.

Immunizing animals with pathogen-specific purified proteins may elicit antibody repertoires that may significantly differ from those generated in the context of the humoral immune responses developed in the course of disease caused by the respective pathogen. As of today, in spite of the diagnostic and therapeutic benefice of a large repertoire of anti-SARS-CoV-2, the isolation of mAbs derived from non-human samples has been documented in few reports [4,25,26]. Specifically, rabbits are considered a major source for a wide variety of monoclonal antibodies with broad utility ranging from clinic diagnosis to human therapy [27,28]. Indeed, it was recently shown that rabbits that were immunized with SARS-CoV-2 spike protein developed a species-specific signature of immunodominant epitopes [29].

The aim of this study was to isolate unique anti-SARS-CoV-2 antibodies in order to expand the existing antibody repertoire, potentially targeting novel epitopes. Based on our experience in rabbit immunization, we employed methodologies that promote high-affinity antibodies [30,31], coupled with efficient screening methods for the isolation of mAbs.

## 2. Materials and Methods

### 2.1. Expression of SARS-CoV-2 Spike Recombinant Protein

Mammalian cell codon optimized sequence, coding for SARS-CoV-2 spike glycoprotein based on the GenPept: QHD43416 ORF [https://www.ncbi.nlm.nih.gov/protein/1791269090]), was used to design pcDNA3.1+-based expression plasmids, mediating recombinant expression of the entire spike glycoprotein (amino acids 1–1207), RBD (amino acids 1–15 and 318–542), NTD (amino acids 1–305) and S1 (amino acids 1–685). The full expression vectors were obtained from Genscript (Piscataway, NJ, USA). A stabilized soluble version of the spike protein was designed by inclusion of the proline substitutions at positions 986 and 987, and disruptive replacement of the furin cleavage site RRAR (residues at position 682–685) with GSAS, as reported [32,33]. C-terminal his-tag, as well as streptag, were included in all constructs in order to facilitate protein purification. The recombinant proteins were expressed in CHO cells using ExpiCHO^TM^ Expression system (Thermo scientific, Waltham, MA, USA) following purification using HisTrap^TM^ (GE Healthcare, Uppsala, Sweden) and Strep-Tactin^®^XT (IBA Lifesciences, Goettingen, Germany). In addition, huFc-RBD and huFC-NTD-fused proteins were expressed using previously designed Fc-fused protein expression vector [34], giving rise to a protein comprising two RBD or NTD moieties owing to the homodimeric human (gamma1) Fc domain (huFc). Expression of the recombinant proteins was performed using ExpiCHO^TM^ Expression system (Thermo scientific) following purification using HiTrap Protein-A column (GE healthcare). All purified proteins were preserved in PBS. The purity of each antigen was evaluated by SDS-PAGE gel under non-reducing conditions.

### 2.2. Rabbits Immunization

Treatment of animals was in accordance with regulations outlined in the U.S. Department of Agriculture (USDA) Animal Welfare Act and the conditions specified in the Guide for Care and Use of Laboratory Animals (National Institute of Health, 2011). Animal studies were approved by the local ethical committee on animal experiments (protocol number Rb-04-20). Female New Zealand white rabbits were maintained at 20−22 °C and a relative humidity of 50 ± 10% on a 12 h light/dark cycle, fed with commercial rodent chow (Koffolk Inc., Tel Aviv, Israel) and provided with tap water ad libitum. Rabbits were subcutaneously (sc) immunized with the 150 µg purified recombinant antigens human Fc-RBD (huFc-RBD), huFc-NTD, S1 or spike S1+S2 (two rabbits per antigen) in complete Freund’s adjuvant (CFA). Starting three weeks post primary immunization, rabbits were boosted twice in incomplete Freund’s adjuvant (IFA) at three week intervals. Terminal bleeding was performed 10 days post the final boost.

### 2.3. ELISA

A standard direct ELISA protocol was applied essentially as described [18]. Microtiter plates were coated using 0.1 μg/well of recombinant SARS-CoV-2 spike, S1 domain, RBD, NTD subunits or huACE2 (Sino Biological, Beijing, China). AP-conjugated anti-rabbit IgG or AP-conjugated anti-human IgG (Jackson ImmunoResearch Laboratories, West Grove, PA, USA) were applied for rabbit sera or recombinant antibodies, respectively. Detection was performed using PNPP substrate (Sigma, Rehovot, Israel). Data points were fitted using non-linear regression (Prism 5, GraphPad, San Diego, CA, USA).

### 2.4. Isolation of Peripheral Blood Mononuclear Cells and Splenocytes

From each group, one rabbit was selected for further analysis and isolation of single B cells by sorting. Whole rabbit blood was mixed 1:1 in phosphate-buffer saline (PBS)+2% fetal calf serum (FCS) and peripheral blood mononuclear cells (PBMCs) were separated using density centrifugation on Ficoll. The PBMCs layer was removed using a pipette and washed in a 10-fold excess (per volume) of PBS+2% FCS by centrifugation at 4 °C, 1200 rpm for 5 min. After removal of the spleen, splenocytes were gently teased out in PBS using forceps with curved ends. Cell clumps were disrupted by pipetting to generate a single-cell suspension. Splenocytes were washed and erythrocytes were lysed by hypotonic shock using RBC Lysis Buffer (BD Pharm Lyse^TM^, San Diego, CA, USA). Following an additional wash, cells were resuspended in PBS+2% FCS and counted by hemocytometer using 0.1% Trypan blue.

### 2.5. Isolation and Ig Gene Amplification of Single B Cells

Approximately 1 × 10^6^ cells were resuspended in PBS+2% FCS and stained with mouse anti-rabbit-IgG-PE (Southern Biotech, Birmingham, AL, USA) and 0.1 μg APC-labelled NTD or S1 using a Lightning Link kit (Innova Biosciences, Cambridge, England) in a final volume of 100 μL. Cells were incubated for 30 min on ice followed by washing and resuspension in 1 mL PBS+2% FCS for FACS sorting by FACSAria III sorter (Becton Dickinson, San Diego, CA, USA), using an Automated Cell Deposition Unit (ACDU). Single B cells were sorted according to surface marker expression patterns IgG^+^/NTD^+^ and IgG^+^/S1^+^ applying FSC-H/FSC-W-based duplet discrimination and single-cell sort mask settings. The cells were sorted into 96-well PCR plates (total of 192 cells for each sort) containing 4 μL/well Lysis solution (0.5 × PBS; 10mM DTT (Invitrogen, Carlsbad, CA, USA); 8 U RNAsin (Promega, Madison, WI, USA); 0.4 U Prime RNAse Inhibitor^TM^ (Qiagen, GmbH, Hilden, Germany)). Plates were sealed and immediately frozen on dry ice before storage at −80 °C. Total RNA from sorted single B cells was reverse transcribed in a final volume of 14 μL/well in the original 96-well sorting plate. A random Hexamer Primer mix solution (3.5 μL/well) containing 1.3 U Prime RNAse Inhibitor; 0.5% v/v Igepal CA-630 (Sigma, Rehovot, Israel); 150 ng Random primer (Invitrogen, Carlsbad, CA, USA) was added into the sorted 96-well plates on ice. The plates were spun down at 4 °C and incubated at 65 °C for 1 min. Reverse transcription (RT) mix solution (7 μL/well) containing 0.1 μL DTT (0.1M); 0.5 μL dNTPs (25 mM each); 5 U Ribonuclease Inhibitor; 0.5 U Prime RNAse Inhibitor; 50 U SuperScript III RT (Invitrogen, Carlsbad, CA, USA) was added to each well on ice. Reverse transcription reactions were performed at 25 °C for 15 min, 42 °C for 5 min, 25 °C for 10 min, 37 °C for 55 min and 94 °C for 5 min. cDNA was stored at −20 °C. Rabbit Igh and Igk V gene transcripts were amplified independently by two rounds of semi-nested PCR, starting from 2 μL of cDNA as template. The first single-cell PCR reaction was performed in 96-well plates containing 2 μL cDNA, 10 μL DreamTaq Mix ×2 (Fermentas, Burlington, Canada), and 200 nM primers. For heavy chain amplification, VH-leader primer 5′-AAGCTTGCCACCATGGAGACTGGGCTGCGCTGGCTTC -3′ [35] and the CIgG outer reverse primer 5′-CCATTGGTGAGGGTGCCCGAG-3′ were used. For kappa L chain amplification, the forward Vκ-leader primer [35] 5′-AAGCTTGCCACCATGGACAYGAGGGCCCCCACTC-3′ and reverse Cκ outter primer 5′-CAGAGTRCTGCTGAGGTTGTAGGTAC-3′ were used. A semi-nested second-round PCR was performed with 1 μL of unpurified first-round PCR product under the same conditions, using the same Igκ and Igh forward primers and the following internal Cκ reverse primer 5′-GGGAAGATGAGGACAGTAGGTGC-3′ or internal CIgG reverse primer 5′-GCAGCAGGGGGCCAG-3′. Both PCR were performed at 94 °C for 5 min, followed by 39 cycles of 94 °C for 30 s, 56 °C for 30 s, 72 °C for 60 s, and finally 72 °C for 5 min. PCR products were analyzed on 2% agarose gels, purified and sent for sequencing. After identification of germline Ig V and J genes by IgBlast, the second PCR reactions were repeated with combinations of gene-specific V and J primers containing restriction sites to allow direct assembly of heavy and light chains into single-chain form following cloning into expression vectors, as previously described [18].

### 2.6. Production of scFv-Fc Antibodies

The desired clones were isolated using QIAprep spin Miniprep kit (Qiagen, GmbH, Hilden, Germany) and the entire scFv was cloned into a pcDNA3.1+-based expression vector that was modified, providing the scFv with the human (IgG1) CH2-CH3 Fc fragments, resulting in scFv-Fc antibody format. ScFv-Fc were expressed using ExpiCHO^TM^ Expression system (Thermo scientific) and purified on HiTrap Protein-A column (GE healthcare).

### 2.7. Cells

VeroE6 cells were obtained from the American Type Culture Collection (ATCC^®^ CRL-1586TM; Summit Pharmaceuticals International). Cells were used and maintained in Dulbecco’s Modified Eagle Medium (DMEM) supplemented with 10% fetal bovine serum (FBS), MEM non-essential amino acids, 2 mM L-Glutamine, 100 Units/mL Penicillin, 0.1 mg/mL streptomycin and 12.5 Units/mL Nystatin (Biological Industries, Beit-Haemek, Israel). Cells were cultured at 37 °C, 5% CO_2_ atmosphere.

### 2.8. Plaque Reduction Neutralization Test (PRNT)

Plaque reduction neutralization test (PRNT) was performed as previously described [36], using SARS-CoV-2 (GISAID accession EPI_ISL_406862, kindly provided by Bundeswehr Institute of Microbiology, Munich, Germany). IC_50_ was defined as mAb concentration or serum dilution, at which the plaque number was reduced by 50%, compared to plaque number of the control (in the absence of Ab/serum). Handling and working with SARS-CoV-2 was conducted in a BL3 facility in accordance with the biosafety guidelines of the Israel institute for biological research (IIBR).

### 2.9. Biolayer Interferometry

Binning studies were carried out using the Octet system (Version 8.1; ForteBio, Fremont, CA, USA) that measures biolayer interferometry (BLI). All steps were performed at 30 °C with shaking at 1500 rpm in black 96-well plates containing 200 μL solution in each well. Streptavidin-coated biosensors were loaded with each of the biotinylated IgG MD29, MD47, MD62 and MD65 antibodies (10 µg/mL) to reach 0.7–1 nm wavelength shift followed by washing. The sensors were reacted for 300 s with monomeric RBD and then transferred to buffer-containing wells for another 60 s (dissociation phase). The antibody-RBD-loaded sensors were then incubated with the non-labeled rabbit derived scFv-Fc mAbs (10 µg/mL). Binding and dissociation were measured as changes over time in light interference after subtraction of parallel measurements from unloaded biosensors. The binding characteristics of the monoclonal antibodies were determined by BLI. Each scFv-Fc mAb was immobilized on a protein A sensor and reacted for 300 s with increasing concentration of monomeric RBD or NTD (association phase) and then transferred to buffer-containing wells for another 300 s (dissociation phase). Sensorgrams (after subtraction of parallel measurements from unloaded biosensors) were fitted with a 1:1 binding model using the Octet data analysis software 8.1.

### 2.10. Epitope Mapping

Lyophilized 240 biotinylated 15 amino-acid long peptides (with 10 amino-acid overlap) covering the entire ectodomain of SARS-CoV-2 spike protein were purchased from JPT Peptide Technologies (Berlin, Germany). All peptides were resuspended in di-methyl sulfoxide (DMSO) to a concentration of 1 mg/mL, aliquoted and stored at −20 °C. An aliquot of the peptides was thawed, diluted 1:100 in 1 × PBS (to reach 10 µg/mL) and added to Maxisorp ELISA plates (Thermo scientific, Waltham, MA, USA) pre-coated with streptavidin and blocked with 2% Bovine serum albumin (BSA). Plated peptides were incubated with the individual monoclonal antibodies (5 µg/mL diluted in blocking buffer) and further incubated with donkey anti-human alkaline phosphatase-conjugated secondary antibody (Jackson ImmunoResearch Laboratories, West Grove, PA, USA). Immune complexes were identified following incubation with SIGMAFAST™ PNPP (Sigma, Rehovot, Israel) and measuring absorbance at 405 nm. For the modeling of mAbs’ recognition sites on SARS-CoV-2 S protein, spike structure with PDB ID 7C2L was used and analyzed by The PyMOL Molecular Graphics System (Version 1.7 Schrödinger, LLC).

## 3. Results

### 3.1. Immunization of Rabbits and Characterization of the Humoral Immune Response

**Figure 1:**
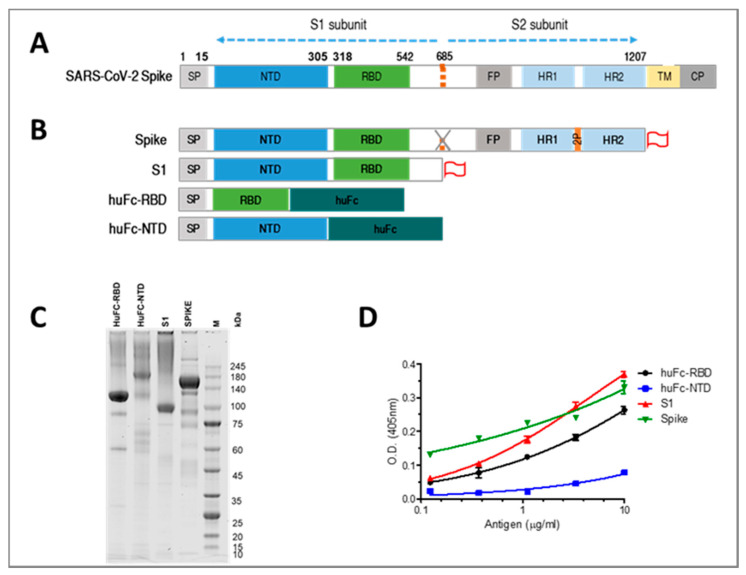
Production and characterization of SARS-CoV-2 spike antigens. Schematic representation of SARS-CoV-2 spike and the recombinant antigens. (**A**) Spike protein consists of a signal peptide (SP) and S1 and S2 subunits. The S1 subunit is composed of the N-terminal domain (NTD) and the receptor binding domain (RBD). The S2 subunit contains the cytoplasm domain (CP), transmembrane domain (TM) and an ectodomain composed of a fusion peptide (FP) and heptad repeats 1 and 2 (HR1 and HR2). (**B**) In the modified spike ectodomain protein, the furin cleavage site located at amino acid 682–685 was replaced for preventing S1 and S2 dissociation and two proline (2P) mutations were added between the HR1 and HR2 regions to stabilize the complex. In addition, a C terminal strep tag and His-tag (represented as red flag) were added to several of the antigens. (**C**) SDS-PAGE analysis under non-reducing conditions of the purified antigens. (**D**) Binding of the purified antigens to huACE2 protein as determined by ELISA. Points are average ± SEM of triplicates.

In order to isolate high-affinity mAbs for diagnosis and therapy, rabbits were immunized by several SARS-CoV-2 spike-related recombinant antigens, an approach which favors elicitation of antibodies recognizing distinct epitopes of the target antigen. Accordingly, four recombinant spike constructs were designed and used for expression of 4 different antigens: (1) spike ectodomain (S1+S2); (2) S1 subunit; (3) human Fc-RBD (huFc-RBD); and (4) huFc-NTD (Figure 1A,B). The antigens were expressed in CHO cells, purified and confirmed to retain their expected molecular weights (Figure 1C). Next, the purified recombinant proteins were verified to maintain the ability to bind human ACE2, indicating that they are properly folded, adopting the spatial structure of the parental viral spike glycoprotein. Indeed, the whole spike ectodomain, the S1 subunit, the huFc-RBD but not the huFc-NTD bound to recombinant human ACE2 in a dose-dependent manner (as established by ELISAs, Figure 1D).

**Figure 2:**
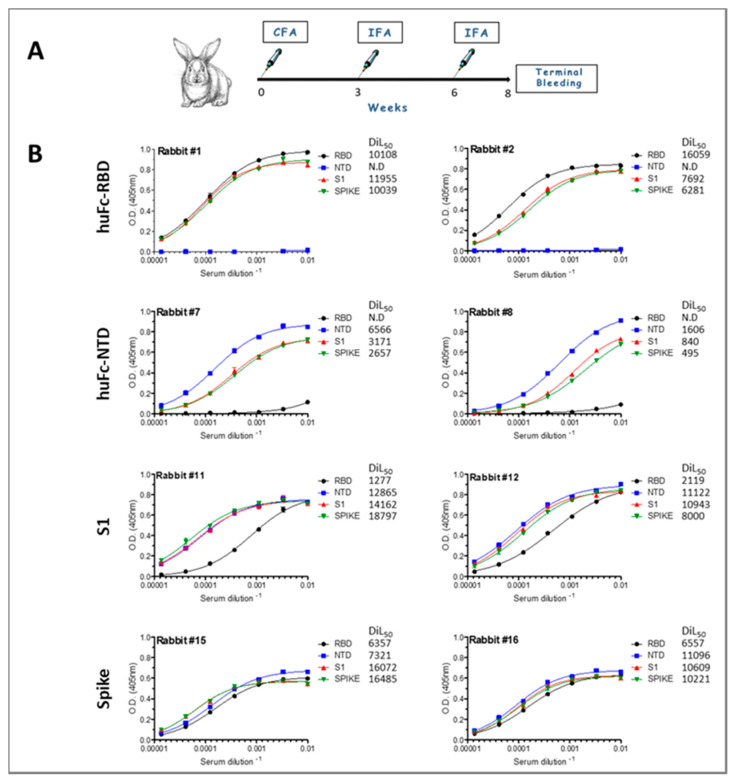
Anti-SARS-CoV-2 IgG antibody responses in immunized rabbits. (**A**) Rabbit immunization regimen scheme. (**B**) Binding curves of IgG polyclonal antibodies from rabbit sera (taken 10 days post the last boost) were obtained by ELISA against the indicated antigens. Antigens that were used to immunize each pair of animals are indicated on the left. Points are average of triplicates ±SEM fitted by non-linear regression and represent two independent experiments. The half-dilution value (DIL_50_) for each antigen is presented.

Next, rabbits (two per group) were subcutaneously (sc) immunized with the purified antigens in complete Freund’s adjuvant (CFA). Starting three weeks post primary immunization, rabbits were boosted twice in incomplete Freund’s adjuvant (IFA) at a three-week interval (Figure 2A). The efficiency of the immunization procedure in eliciting antibody responses was monitored by ELISA, 10 days after the final boost. Indeed, the majority of the immunized rabbits developed a potent antibody response towards the administered antigen, with a serum half-dilution value (DIL_50_) > 6500 (Figure 2). The only exception is rabbit 8 that was immunized with huFc-NTD and developed a moderate antibody response (DIL_50_ of 1600). To further verify the specificity of the elicited antibodies in each immunization protocol, binding assays were performed for each rabbit sera against RBD, NTD, S1 and the spike. Animals that were immunized with either the RBD or the NTD developed specific antibody responses that cross-reacted with the spike protein and the S1 subunit. It was also found that animals that were immunized with either the spike or the S1 subunit developed strong antibody responses toward all tested antigens, with significant antibody binding toward the NTD compared to the RBD. These results suggest that in this format the RBD is somewhat less immunogenic, especially when compared to the response obtained using the RBD as the antigen.

**Figure 3:**
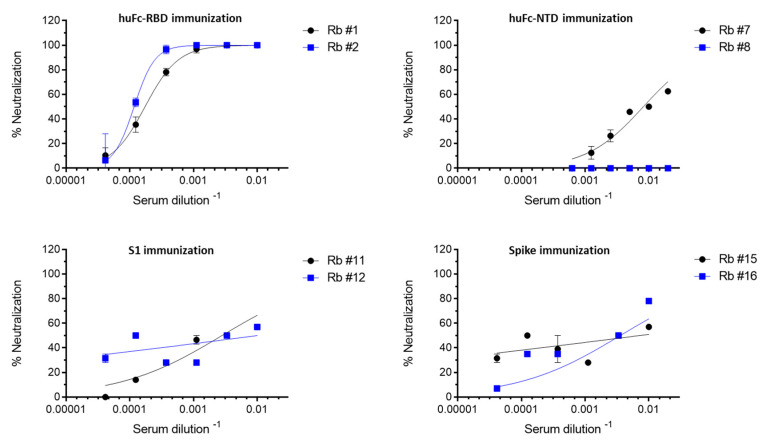
SARS-CoV-2 neutralization activity of immunized rabbits’ sera. Serum neutralization activity against SARS-CoV-2 was evaluated by the ability to reduce plaques formation. Results are expressed as percent inhibition of control without Ab. Points are average of duplicates ±SEM fitted by non-linear regression and represent two independent experiments obtaining similar results.

To measure the neutralizing activity of the sera obtained from immunized rabbits, a plaque reduction neutralization test (PRNT) was performed using VeroE6 cells infected with authentic SARS-CoV-2. The neutralizing potency of each sera was calculated as the dilution that reduced plaque formation to 50% (NT_50_) compared to control. Highly neutralizing effect (NT_50_ 5800–8600) was observed for the sera collected from huFc-RBD-immunized rabbits whereas only a limited neutralization effect was observed for the sera collected from rabbits following spike and S1 immunization (NT_50_ of ~450 and ~350, respectively, Figure 3). This observation demonstrates that the RBD immunogen by itself elicited the major source for high affinity and neutralizing antibodies. In addition, immunization with NTD correlated with the titer of anti-NTD antibodies, as evidenced by the observation that rabbit 7 displayed an NT_50_ of 120, whereas rabbit 8 had no detectable neutralizing activity. These results are in line with previous reports showing that vulnerable epitopes located within non-RBD regions of the spike can serve as alternative and complementary targets for SARS-CoV-2 neutralization [8,20,21,22].

### 3.2. Isolation of Anti-RBD Antibodies

**Figure 4:**
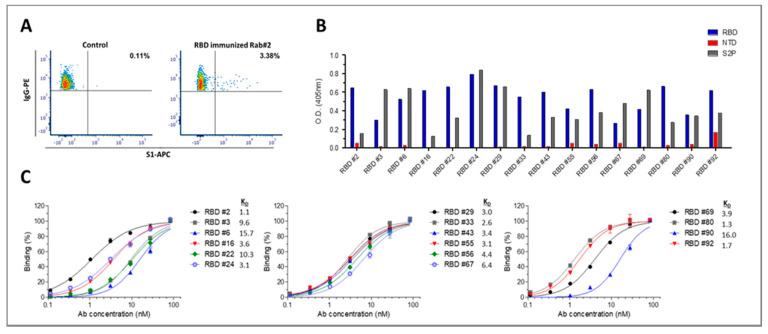
Isolation and characterization of anti-RBD antibodies. (**A**) Splenocytes were isolated from huFc-RBD-immunized rabbit 2 and were analyzed by flow cytometry using anti IgG-PE and APC Figure 1. IgG^+^/APC-S1^+^ B cells (located at the upper right quadrant and represented as percent out of total IgG^+^ cells) were single-cell sorted into 96-well microplates by FACSAria. Naïve rabbit PBMCs were analyzed as a negative control, demonstrating the specificity of the sorting strategy. (**B**) Specificity of the anti-RBD antibodies was determined by ELISA against the indicated SARS-CoV-2 proteins. Results are averages of duplicates from a representative experiment (out of two performed with similar results). (**C**) Binding profile of the antibodies determined by ELISA using RBD as the coating antigen. Data are presented as binding percent of B_max_ for each antibody. The values represent average of triplicates ±SEM fitted by non-linear regression. The apparent K_D_ (nM) value of each antibody is shown.

In order to expand the repertoire of anti-SARS-CoV-2 mAbs directed against RBD and non-RBD-related epitopes, a single B cell sorting strategy was applied. Splenocytes harvested from huFc-RBD-immunized rabbit 2, which has shown the highest binding and neutralizing properties, were analyzed by flow cytometry for targeting specific anti-RBD IgG class-switched memory B cells. Three percent of the total IgG^+^ cells bound to fluorescent-labeled S1 (Figure 4A). Out of these, single-cell clones were sorted and 42 anti-RBD sorted B cells were found to carry unique sequences. To further characterize the antibodies generated by these cells, the VH/VL RNA sequences were amplified, cloned and expressed as scFv-Fc antibodies. Half of the unique clones (21 out of 42) expressed antibodies that bound S1, indicating the high specificity of the sorting strategy.

To further characterize the specificity of the anti-RBD antibodies, the top 16 antibodies exhibiting the highest binding toward S1 were tested for their ability to bind the spike ectodomain, RBD and NTD. All selected antibodies specifically interacted with the RBD-containing antigens with no or minimal binding to NTD (Figure 4B). Half maximal binding ELISA test for these antibodies toward RBD was performed, and apparent K_D_ values of 1.1–16 nM were determined (Figure 4C). To further validate the binding data, we have performed a more detailed analysis to selected antibodies using biolayer interferometry (BLI), that resulted in similar affinity values (0.7–7 nM; Supplementary Table S1).

### 3.3. Isolation of Anti-NTD Antibodies

**Figure 5:**
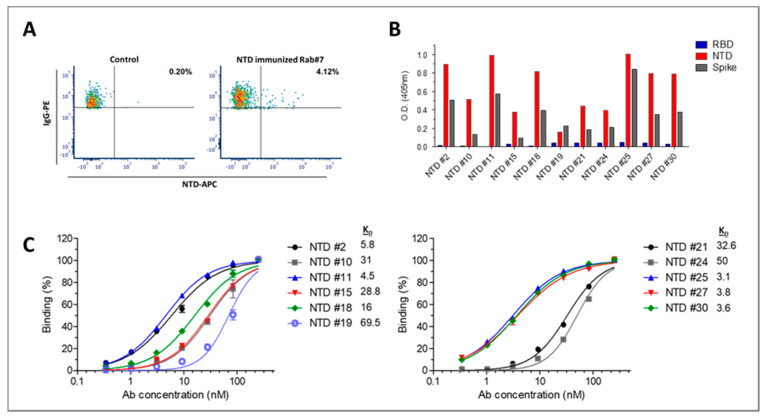
Isolation and Characterization of anti-NTD antibodies. (**A**) Splenocytes were isolated from huFc-NTD-immunized rabbit 7 and were analyzed by flow cytometry using anti IgG-PE and APC fluorescence conjugated antigens NTD. IgG^+^/APC-NTD^+^ B cells (located at the upper right quadrant and represented as percent out of total IgG+ cells) were single-cell sorted into 96-well microplates by FACSAria. Naïve rabbit PBMCs were analyzed as a negative control, demonstrating the specificity of the sorting strategy. (**B**) Specificity of the anti-NTD antibodies was determined by ELISA against the indicated SARS-CoV-2 proteins. Results are averages of duplicates from representative experiment (out of two performed with similar results). (**C**) Binding profiles of the antibodies determined by ELISA using NTD as the coating antigen. Data presented as binding percent of B_max_ for each antibody. The values represent average of triplicates ± SEM fitted by non-linear regression. The apparent K_D_ (nM) value of each antibody is shown.

We next sought to isolate antibodies directed against non-RBD epitopes, which may also have the potential to neutralize the virus. The fact that rabbit 7, that was immunized with NTD, exhibited significant SARS-CoV-2 neutralizing activity, indicated that lymphocytes collected from this rabbit may serve as a good source for isolating such antibodies. Thus, flow cytometry for targeting specific anti-NTD IgG class-switched memory B cells was applied using a fluorescently labeled NTD (Figure 5A). Unique antibody sequences from 26 cells isolated by cell sorting were further cloned as a scFv-Fc format, of which 13 antibodies were found to bind S1. From this panel, 11 antibodies (with the highest binding properties against S1) were found to specifically bind NTD (Figure 5B). Analysis of the antibody binding profiles toward NTD enabled their classification into two affinity groups, the top 5 antibodies with apparent K_D_ values of 3–6 nM and the rest exhibiting apparent K_D_ values of 16–70 nM (Figure 5C). The top three antibodies were also characterized using BLI analysis that resulted in affinity values of 0.6, 7 and 16 nM for antibodies NTD25, NTD30 and NTD27, respectively (Supplementary Table S1). Over all, the results so far indicate the successful isolation of antibodies directed to RBD and NTD with high specificity and affinity.

### 3.4. Neutralizing Potency of the Selected mAbs

**Figure 6:**
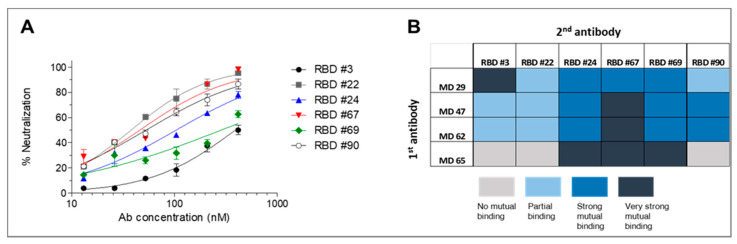
SARS-CoV-2 neutralization and epitope binning. (**A**) SARS-CoV-2 in vitro neutralization was determined by the ability of each antibody to reduce plaques formation. Results are expressed as percent inhibition of control without Ab. The values are average of duplicates ± SEM fitted by non-linear regression and represent three independent experiments showing similar results. (**B**) Epitope binning of rabbit-derived antibodies was evaluated by the ability of each antibody to simultaneously bind RBD with four human-derived neutralizing antibodies directed to four distinct epitopes within the RBD.

The potential of the individual antibodies to neutralize SARS-CoV-2 was further evaluated by the plaque reduction neutralization test (PRNT) using VeroE6 cells. It was found that 6 out of 16 antibodies directed against the RBD were able to neutralize the virus, exhibiting NT_50_ values ranging from 40 nM (antibodies 22 and 67) and up to 400 nM (antibody 3, Figure 6A). No correlation between affinity and neutralizing activity was observed for the six neutralizing antibodies. None of the anti-NTD antibodies exhibited neutralization activity.

### 3.5. Epitope Mapping

Classification of antibodies based on their specific targeted antigenic epitopes allows the integration of several non-competing antibodies in diagnosis and therapy procedures, potentially enabling improved pathogen capture and neutralization as well as mitigating the emergence of immune escape mutants. Recently, we reported the isolation of human neutralizing antibodies (MD29, MD47, MD62 and MD65) [18] that target four distinct epitopes within the RBD. It was therefore of interest to determine whether the novel anti-RBD neutralizing antibodies documented in the current report bind to the same or to different epitopes. Accordingly, biolayer interferometry (BLI) epitope binning was performed. Biotinylated human-derived antibodies were used to capture the RBD and the antibody-RBD complexes were then reacted with each of the novel antibodies. Simultaneous binding of the rabbit-derived antibody to RBD induces a wavelength shift whereas if the two antibodies bind the same or partially overlapping RBD epitope, no or very low wavelength shift, respectively, is expected. It was found that antibodies 3, 22 and 90 could not bind to the MD65-RBD complex (Figure 6B and Supplementary Figure S1). Interestingly, antibodies 24, 67 and 69 could bind to the RBD that was pre-associated with all MD antibodies, thus indicating the existence of a unique epitopes.

**Figure 7:**
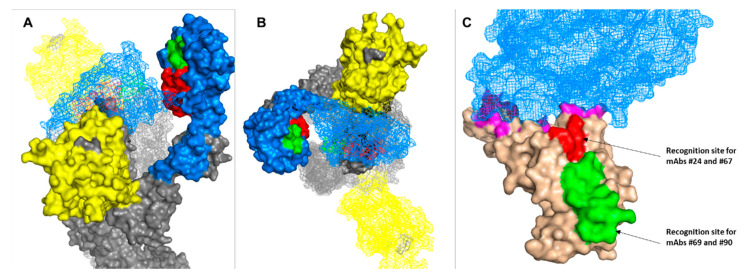
Modeling of the epitopes targeted by anti-RBD antibodies. Side (**A**) and top (**B**) view of epitope ^404^GDEVRQI^410^ (green: recognition site for antibodies #69 and #90) and epitope ^376^TFKCYGVSPTKLNDL^390^ (red: recognition site for antibodies #24 and #67) localization on two out of the three subunits composing the crystal structure of SARS-CoV-2 spike (PDB 7C2l). The subunit whose RBD (light blue) is in the “up” configuration is presented in surface mode, whereas the other subunit whose RBD is in the “down” formation is shown as a mesh. The NTD is colored in yellow. (**C**) Crystal structure (PDB 6lzg) of RBD (light brown) bound to ACE2 (light blue mesh). RBD residues that are in direct contact with the receptor are colored in magenta. Neutralizing antibodies’ epitopes are colored in green and red.

To further characterize the epitopes that each of the antibodies recognizes, the binding of the six antibodies to an overlapping 15-mer peptide array that covers the RBD of SARS-CoV-2 spike (amino acids 316-550) was examined. It was found that antibodies 24 and 67 specifically bind an epitope spanning amino acids ^376^TFKCYGVSPTKLNDL^390^ and antibodies 69 and 90 bind to an adjacent epitope located between amino acid ^396^YADSFVIUGDEVRQI^410^ (Figure 7A,B). In contrast, antibodies 3 and 22 did not bind any of the peptides, suggesting that they target non-linear epitopes possibly defined by the spatial architecture of the protein. Such epitopes are inherently non-detectable by the peptide array.

Visualization of the second epitope (recognized by Ab 69 and 90) on the crystal structure of the spike (Figure 7) may allow to speculate that the actual epitope can be further narrowed to include only amino acid ^404^GDEVRQI^410^, as the first 8 amino acids of the peptide are not exposed to the surface of the Spike trimer. Furthermore, it appears that this epitope is accessible to the neutralizing antibody only if RBD adopts the “up” conformation. By the same line of reasoning, the epitope that is recognized by antibodies 24 and 67 is “buried” between the three RBDs and thus may be accessible to the neutralizing antibodies only if two or all receptor binding domains adopt the “up” conformation. Positioning the two epitopes on the structure of the spike trimer suggests that they do not overlap with the ACE2 binding motif (Figure 7C).

## 4. Discussion

The recent emergence of different variants of SARS-CoV-2 established that the evolution of mutations, which may compromise the efficacy of existing COVID-19 immunodiagnostics or immunotherapies, is a tangible possibility of tremendous public health concern. One of the approaches to circumvent this complication is expansion of the repertoire of diagnostic and therapeutic antibodies that target distinct (vulnerable) epitopes.

Most of the highly neutralizing antibodies reported against SARS-CoV-2 were derived from convalescent individuals and shown to target the RBD [15,16,17,18,19,37,38,39]. Recently, vulnerable epitopes located within non-RBD regions were also shown to be the target of several SARS-CoV-2 neutralizing antibodies. Specifically, the N-terminal domain (NTD) of the S1 subunit [8,20,21,22] and the S2 region [23] were suggested as an alternative and/or complementary target for neutralizing antibodies. 

In this study, we utilized targeted vaccination with spike antigens in rabbits. This allowed the elicitation of antibodies directed to vulnerable epitopes or those having relatively low immunogenicity that are not routinely exposed to the immune system in the course of the infection. We successfully isolated a set of anti-RBD and anti-NTD-specific mAbs using a single-cell sorting strategy. These antibodies were characterized as having high specificity and affinity profiles. Moreover, anti-RBD antibodies showed an in vitro neutralizing potency against SARS-CoV-2 directed to a novel epitope within the RBD.

The humoral response profile of the immunized rabbits indicated that the high binding capacity determined against the different antigens used for immunization did not necessarily correlate with the resulting neutralization potency. The immunization with the spike ectodomain and the S1 elicited fewer neutralizing antibodies as RBD. This observation confirms previous reports demonstrating that the RBD immunogen by itself elicited the major pool of high affinity and neutralizing antibodies in a universal species-independent manner, while the immunogenicity of the spike and its counterpart S1 and S2 subunits vary depending on animal model, antigen structure and the immunization procedures [29,40,41,42].

Two linear epitopes were identified in the current work as the target of anti-RBD neutralizing antibodies. One of these epitopes (targeted by antibodies 24 and 67) was previously found to be the target of a human-derived anti-SARS-CoV-2 antibody [43]. However, to the best of our knowledge, the second linear epitope (^404^GDEVRQI^410^) targeted by antibodies 69 and 90 is novel and has not been previously described. This epitope is part of the η3 loop which is part of the regions that construct a cavity on one side of the RBD. It was previously shown that residue V^408^ is part of a large, non-linear epitope of another SARS-CoV-2 neutralizing antibody [44], thus our results further emphasize the role of this region in virus neutralization.

The fact that antibodies 69 and 90 bind to the same epitope is in apparent disagreement with the observation that antibody 90 does not compete with antibody MD65, whereas antibody 69 does. A possible explanation to this apparent inconsistency is that the linear epitope recognized by both antibodies adopts a different conformation upon binding to either antibody, one of which induces steric interference with antibody MD65.

It was previously reported that SARS-CoV-2 antibody-mediated neutralization does not necessarily require the abrogation of the RBD–ACE interaction [18,25]. Indeed, positioning the two epitopes on the structure of the spike trimer suggests that they do not overlap with the ACE2 binding motif. The exact mechanism of neutralization mediated by the novel antibodies will require further elucidation.

One of the goals of the current work was the isolation of antibodies targeting non-RBD moieties. As it was shown recently, monoclonal neutralizing antibodies targeting the NTD were successfully isolated from blood samples of COVID-19 patients or convalescent individuals, attesting to the important role of this domain in the virus pathogenicity [8,20,21,22]. Here, we found that rabbits that were immunized with NTD or an NTD-containing protein (S1 and spike) developed high titers of NTD-binding antibodies. However, the neutralizing titers of these animals were significantly lower than the RBD-immunized animals. Moreover, the whole panel of anti-NTD monoclonal antibodies isolated here lacked any neutralizing activity. These results are in good agreement with a recent study in which it was shown that non-human primates immunized with Fc-NTD developed high titers of binding antibodies, yet were devoid of neutralizing capacity ability [45]. The discrepancy between the two types of responses (human versus immunized animals) may stem from selective pressure on the immune system during the course of the disease, leading to favoring elicitation of antibodies against vulnerable viral epitopes. When immunization is carried out with purified antigens, the immune system may develop antibodies according to the immunogenic properties of the epitopes, which do not necessarily correlate with neutralization capacity. It is possible that by mapping the immunodominant epitopes in the sera of the NTD-immunized animals compared to parallel studies mapping COVID-19 convalescent sera samples, a targeted immunization strategy enabling the elicitation of NTD-specific neutralizing antibodies could be devised.

Novel SARS-CoV-2 variants continuously emerge throughout the world, containing multiple mutations in the spike glycoprotein. While numerous mAbs were shown to effectively neutralize the original virus, the activity of many antibodies was challenged or complicated by these variants [46,47,48,49,50]. It would be interesting to assess the binding and activity characteristics of our novel antibodies towards selected variants and to define the best antibody combinations.

In conclusion, the current study documents the successful identification and recombinant production of a set of anti-RBD and anti-NTD-specific mAbs isolated by a single-cell sorting strategy of lymphocytes collected from rabbits immunized with different spike-derived antigens. These antibodies were characterized as having high specificity and affinity profiles. Anti-RBD antibodies showed in vitro neutralizing potency against SARS-CoV-2 directed to a novel epitope within the RBD. These antibodies may represent the basis for future development of immunodiagnostics and immunotherapy.

## Data Availability

Antibodies are available (by contacting Ohad Mazor from the Israel Institute for Biological Research; ohadm@iibr.gov.il) for research purposes only under an MTA, which allows the use of the antibodies for non-commercial purposes but not their disclosure to third parties. All other data are available from the corresponding authors upon reasonable requests.

## Supplementary Materials

The following are available online at https://www.mdpi.com/article/10.3390/v13040566/s1, Figure S1: Epitope binning of antibodies determined by BLI analysis. Table S1: Binding characteristics of the monoclonal antibodies determined using biolayer interferometry.
