# Supplementary material for: Neutralizing Monoclonal Anti-SARS-CoV-2 Antibodies Isolated from Immunized Rabbits Define Novel Vulnerable Spike-Protein Epitope
Source: Viruses. 2021 Mar 26;13(4):566. doi: 10.3390/v13040566 (PMC8065470; doi:10.3390/v13040566)
Supplement: Supplementary file 1 [file viruses-13-00566-s001.pdf]

Makdasi et al, 2021

Supplementary Table 1

Supplementary Table 1. Binding characteristics of the monoclonal antibodies determined using biolayer interferometry

Ab	K_{on} (1/Ms)	K_{off} (1/s)	K_D (M)
**RBD 2**	$6.0x10^{4}$	$2.9x10^{-4}$	$4.7x10^{-9}$
**RBD 3**	$5.4x10^{4}$	$3.6x10^{-4}$	$6.6x10^{-9}$
**RBD 16**	$9.1x10^{4}$	$4.5x10^{-4}$	$5.0x10^{-9}$
**RBD 24**	$2.5x10^{5}$	$1.7x10^{-4}$	$6.6x10^{-10}$
**RBD 33**	$1.1x10^{5}$	$8.1x10^{-5}$	$6.9x10^{-10}$
**RBD 67**	$1.8x10^{5}$	$1.1x10^{-3}$	$5.9x10^{-9}$
**RBD 69**	$1.1x10^{5}$	$1.5x10^{-4}$	$1.3x10^{-9}$
**RBD 80**	$8.4x10^{4}$	$1.5x10^{-4}$	$1.8x10^{-9}$
**NTD 25**	$1.6x10^{5}$	$9.8x10^{-5}$	$6.0x10^{-10}$
**NTD 27**	$3.0x10^{4}$	$4.9x10^{-4}$	$1.6x10^{-8}$
**NTD 30**	$3.4x10^{4}$	$2.4x10^{-4}$	$7.0x10^{-9}$

<u>**Supplementary Figure 1**</u>

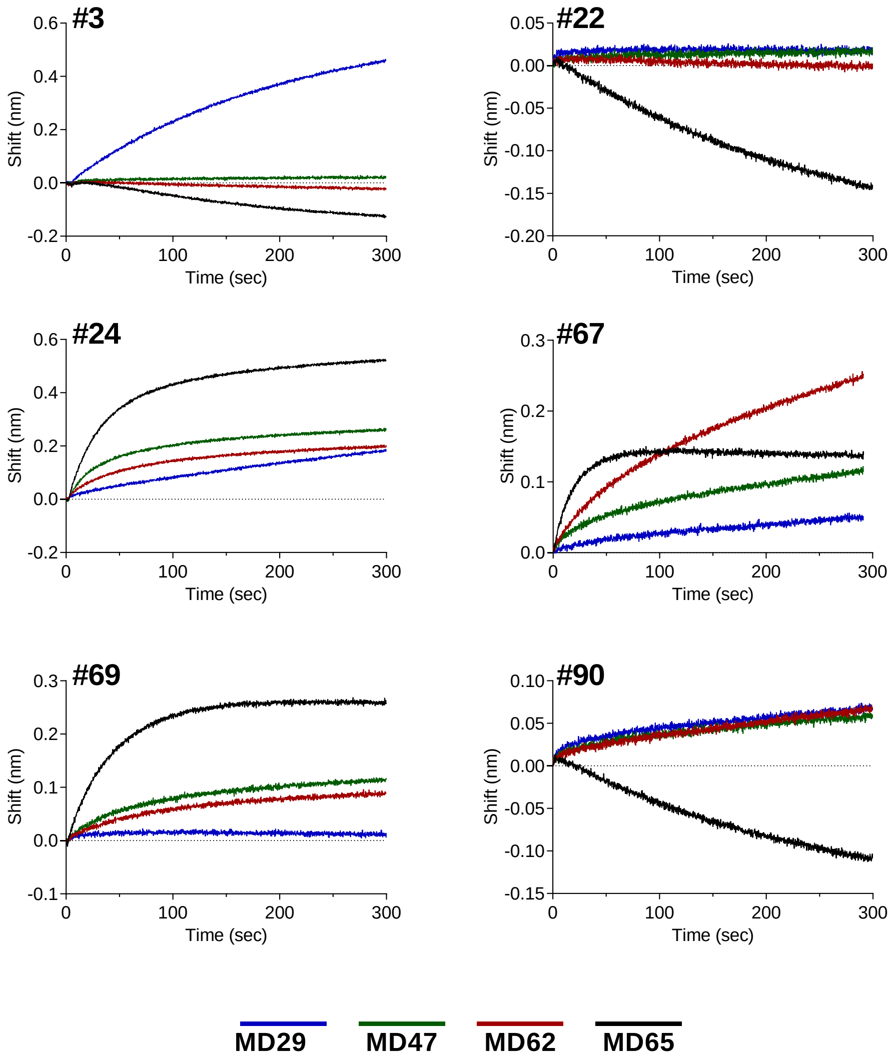

Epitope binning of antibodies determined by BLI analysis. Epitope binning of rabbit-derived antibodies was evaluated by the ability of each antibody to simultaneously bind RBD with four human-derived neutralizing antibodies directed to four distinct epitopes within the RBD. Streptavidin-coated biosensors were loaded with biotinylated MD29, MD47, MD62 or MD65 antibody and reacted for 300 seconds with the monomeric RBD, washed and then incubated with the indicated antibodies for another 300 seconds. The last step is presented for each indicated streptavidin-coated biosensors. In each set of experiments, the background signal was obtained from a parallel sensor incubated with the homologous antibody and sensograms are presented after subtraction of the background signal.
